# 4117 UNIQUE VAGINAL MICROBIOME POPULATIONS AND MICROBIAL GENE CONTENT AMONG WOMEN WHO NATURALLY CONTROL HIV PROGRESSION

**DOI:** 10.1017/cts.2020.102

**Published:** 2020-07-29

**Authors:** Katherine Gisella Michel, Bing Ma, Kathleen Weber, Leah McClellan, Anandi Sheth, Stephen Gange, Audrey French, Jacques Ravel, Igho Ofotokun, Daniel Merenstein

**Affiliations:** 1 Georgetown - Howard Universities

## Abstract

OBJECTIVES/GOALS: The role of the vaginal microbiome (VM) in HIV disease progression is poorly understood. We examined VMs of HIV+ Elite Controllers (ECs) and HIV+ Long-Term Non-Progressors (LTNPs) compared to controls: HIV-positive antiretroviral (ARV) treated (HIV+ATs) and HIV-negative women in the Women’s Interagency HIV Study (DC/Chicago/Atlanta sites). METHODS/STUDY POPULATION: VMs were surveyed via both V3/V4 region of 16S rRNA gene amplicon sequencing and metagenomics sequencing in 67 women across 4 study groups: 1) LTNPs (CD4 >500 cells/mL for 5+ years without ARVs) (n = 7) and 2) ECs (HIV RNA <80 copies/mL for 2+ years without ARVs) (n = 8), matched with 3) HIV+ ATs (on ARVs for ≥1 year with CD4 increase ≥100 cells/mm^3^) (n = 34), and 4) HIV- women (n = 18). Metagenomes were characterized from specimens collected at two time points: 1) vaginal swabs collected 2016-2017 (n = 62) and 2) cervicovaginal lavage collected 2002-2016 (n = 35; DC/Chicago only). We used VIRGO (human vaginal non-redundant gene catalog), a newly developed referencing framework to comprehensively catalog VM gene content, taxonomy and functions. RESULTS/ANTICIPATED RESULTS: Women were 89% African American with a mean age of 46 years (SD 8.8). The most prevalent species were *Gardnerella vaginalis* (predominant in 34%), *Lactobacillus iners* (predominant in 21%), and *L. crispatus* (predominant in 14%). 90% of LTNP and 45% of EC samples were *Lactobacillus*-dominant vs. 28% of HIV- and 30% of HIV+ATs. *L. crispatus* and *L. iners* in ECs/LTNPs had significantly different gene content and greater gene richness vs. controls. *G. vaginalis*-predominant communities were found in 66% of HIV- and 68% of HIV+ATs, compared to 46% of EC and 0% of LTNP. The *G. vaginalis* strains present in EC/LTNP also showed significantly lower gene richness and different gene content vs. controls. DISCUSSION/SIGNIFICANCE OF IMPACT: These results suggest unique VM communities among EC/LTNP, and led us to hypothesize that differential regulation of vaginal immunity drives the observed differences. The similarity between VMs of HIV- and HIV+ATs warrants further study. Larger longitudinal VM studies are needed to assess associated functional pathways and understand the etiology of VM association with HIV progression. CONFLICT OF INTEREST DESCRIPTION: The authors have no conflicts of interest to disclose.

